# Effects of permafrost collapse on soil bacterial communities in a wet meadow on the northern Qinghai-Tibetan Plateau

**DOI:** 10.1186/s12898-018-0183-y

**Published:** 2018-08-22

**Authors:** Xiaodong Wu, Haiyan Xu, Guimin Liu, Lin Zhao, Cuicui Mu

**Affiliations:** 1 Cryosphere Research Station on the Qinghai-Tibetan Plateau, State Key Laboratory of Cryospheric Sciences, Northwest Institute of Eco-Environment and Resource, Chinese Academy of Sciences, Lanzhou, 730000 Gansu China; 2 School of Environmental and Municipal Engineering, Lanzhou Jiaotong University, Lanzhou, 730070 China; 3 Key Laboratory of Western China’s Environmental Systems (Ministry of Education), College of Earth and Environmental Sciences, Lanzhou University, Lanzhou, 730000 China

**Keywords:** Permafrost degradation, Thermokarst, Relative abundance, Organic carbon, Depth, Illumina sequencing

## Abstract

**Background:**

Permafrost degradation may develop thermokarst landforms, which substantially change physico–chemical characteristics in the soil as well as the soil carbon stock. However, little is known about changes of bacterial community among the microfeatures within thermokarst area.

**Results:**

We investigated bacterial communities using the Illumina sequencing method and examined their relationships with soil parameters in a thermokarst feature on the northern Qinghai-Tibetan Plateau. We categorized the ground surface into three different micro-relief patches based on the type and extent of permafrost collapse (control, collapsing and subsided areas). Permafrost collapse significantly decreased the soil carbon density and moisture content in the upper 10 cm samples in the collapsing areas. The highest loading factors for the first principal component (PC) extracted from the soil parameters were soil carbon and nitrogen contents, while soil moisture content and C:N ratios were the highest loading factors for the second PC. The relative abundance of Acidobacteria decreased with depth. Bacterial diversity in subsided areas was higher than that in control areas.

**Conclusions:**

Bacterial community structure was significantly affected by pH and depth. The relative abundance of Gemmatimonadetes and Firmicutes were significantly correlated with the first and second PCs extracted from multiple soil parameters, suggesting these phyla could be used as indicators for the soil parameters in the thermokarst terrain.

**Electronic supplementary material:**

The online version of this article (10.1186/s12898-018-0183-y) contains supplementary material, which is available to authorized users.

## Background

Permafrost accounts for about one quarter of land surface in the northern hemisphere. Approximately 1035 ± 150 Pg soil organic carbon (SOC) was stored in the upper 3 m soils in the circum-arctic permafrost regions [1]. The dynamics of these carbon pools can greatly change greenhouse gas concentration in the air. Permafrost degradation can result in greenhouse gas emissions and further potentially create a positive feedback to climate warming [2]. The permafrost degradation not only stimulates decomposition of soil organic matter (SOM) due to the increasing soil temperature [3], it also affects SOC dynamics via several mechanisms such as changes in vegetation growth, dissolved organic carbon transport, and even the landform [4]. Among these mechanisms, the landform change, i.e., thermokarst formation is one of the most recognizable ground features [5]. Thermokarst greatly changes soil physico–chemical characteristics, as well as the SOC content and biodegradability [6], and thus was considered as a major source of uncertainty in predicting permafrost carbon balance [2]. Therefore, understanding the changes soil parameters and biogeochemistry in thermokarst landforms is a critical step toward the evaluation of permafrost carbon cycle.

Soil bacteria are important drivers for almost all biogeochemical cycles including that SOM decomposition in soils [7]. In permafrost regions, proteobacteria are usually the dominant phyla, and bacterial communities are sensitive to many biotic and abiotic factors such as pH [8, 9], soil moisture content [10], nutrients [11] and organic carbon content [12]. Since thermokarst changes soil moisture, temperature, pH [13], and decreases carbon and nitrogen stocks, as well as their chemical characteristics [14], it would be reasonable to expect that thermokarst has great impacts on bacterial community structure. Although the importance of effects of thermokarst on microbes has been raised several years ago [15], reports to the bacterial community in thermokarst terrains were mainly focused on the thermokarst ponds [16, 17]. In these aquatic ecosystems, proteobacteria were also the dominant phyla and the bacterial community was affected by factors such as pH, dissolved oxygen, dissolved organic carbon [16, 17]. In addition to the thermokarst ponds [18], there are several typical thermokarst modes such as thaw subsidence, permafrost collapse in the terrestrial ecosystems [18]. It has been indicated that the vertical distribution pattern of bacteria has been disturbed and showed a mixed pattern in a permafrost thaw induced subsidence area [19]. However, little is known about the bacterial communities within thermokarst terrains which usually associate with removal of surface soil layers in terrestrial ecosystems.

The Qinghai-Tibetan Plateau (QTP) is the largest high-latitude permafrost region in the world. The QTP, also called “roof of the world”, affects hydrothermal circulations over Asian and even the globe. The permafrost carbon pools are important for quantifying the regional and even global carbon cycle [20–22]. In comparison with the circum-Arctic regions, permafrost on the QTP is more vulnerable to global warming due to its high temperature thermal regime [23, 24], and the thaw slump, permafrost collapse has been widely recorded on the QTP [25]. Based on the knowledge that pH, SOC, and C:N ratios are the most important factors driving soil bacterial community [12] and they vary considerably in thermokarst landforms [26], we hypothesize that permafrost collapse has great effects on bacterial communities. Specifically, we hypothesize that (1) the relative abundances of the dominant phyla showed different patterns within the microfeatures in permafrost collapse area; (2) the changes of pH, SOC, and C:N ratios regulated soil bacterial communities in permafrost collapse-affected area; (3) the relative abundance of specific bacterial phyla showed significant changes along with the soil parameters. Since the proteobacteria are often dominant in many soils in permafrost regions, the abundancies of specific bacterial phyla can be used as indicators for the changes in soil physico–chemical characteristics. To test these hypotheses, we collected soil samples from a permafrost collapse feature on the northern QTP [27, 28] to investigate the bacterial communities and also examine their relationships with soil parameters.

## Methods

### Site description and soil sampling

The study area is located in a permafrost region on the northern QTP (100.913°E, 38.001°N, 3661 m). The land cover is a typical alpine wet meadow, with predominant species of *Kobresia tibetica Maxim*., *K. pygmaea*, and *K. humilis*. According to soil taxonomy [29], the soil type is classified as Typic Haploturbel (ABGE).

**Fig. 1:**
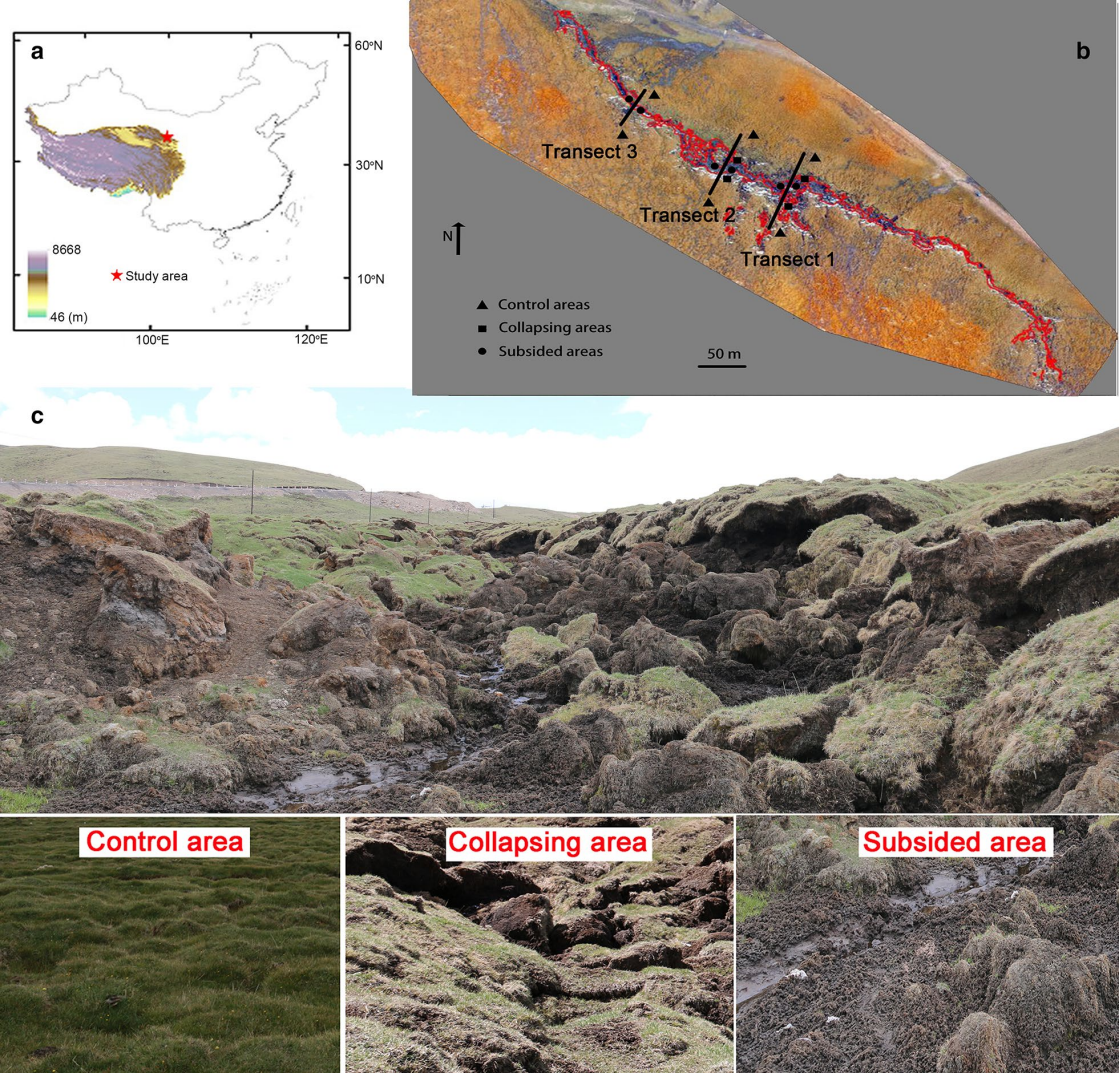
Location of the study area (**a**) and sampling sites (**b**) for the three stages of permafrost collapse areas (control, collapsing, and subsided). The red line denotes the permafrost collapse-affected area. The bottom image shows (**c**) the three different micro-topographies

The area is experiencing an active thermokarst process and there is a thermokarst gully, with a width of about 15 m. The thawed area expands laterally upslope along the exposed headwall, and thus it is similar with thermal erosion in permafrost regions. However, there was little water flow in this gully. According to the thermokarst modes [30], this landform was more likely retrogressive thaw slumps and thus we defined it as permafrost collapse. According to the land surface characteristics, we defined three stages of the permafrost collapse: control areas (C), collapsing areas (D), and subsided areas (S) (Fig. 1). We selected three transects at the intervals of 100 m for sample collection along with the gully. The active layer (surface soil layer that thaws during warm season and freezes cold season) thickness varied from 70 to 120 cm across the microtopography. The thaw depths (which are less than active layer thickness since the active layer is the maximum thaw depth in late September) measured using a steel probe were 110 cm and 50 cm in the control and subsided areas. In the collapsing areas, the thaw depths varied from 70 to 100 cm due to the uneven ground surface. Soil samples were collected at depths of 0–10, 10–25, 25–35, and 35–50 cm at the three stages, and all these samples were taken from the active layers and were not frozen during the sample collection.

The soil samples were collected aseptically using ethanol-disinfected soil augers (3.5 cm in diameter) in the August 2015. At each stage, we selected 1 m × 1 m plots selected to collect the soil samples. The control areas were about 10 m away from the gully, and the collapsing areas were located in the edges of the gully. The subside areas were located in the middle of the subsided area (about 7 m away from the edge of the gully). One transect has narrow boundary area, and the collapsing edge is sharp, it was difficult to collect samples from the collapsing stage, and only samples from control and subsided areas were collected (Fig. 1). For the other two transects, we collected the soil samples for all the three stages. Since there are considerable heterogeneities in soil properties within the same plots in most soils, there is a possibility that the samples used for DNA extraction were not exactly same as the samples for physico–chemical properties measurement. Therefore, we mixed five soil cores in a sterile bag to produce a sample, then we divided this mixed sample into three subsamples. This method ensured that the soil parameters and sequencing data were from same samples, although standard errors in the soil samples were measurement errors. The samples were placed in clean, sealable plastic bags, transported immediately to the laboratory in a cooler with ice packs, and stored at − 80 °C until genomic DNA extraction was carried out.

### Soil parameters measurement and soil DNA extraction

Soil moisture content was determined by drying the soil at 105 °C for 8 h. The pH and conductivity of a soil suspension (1:5 soil:water ratio) were measured with an acidity meter (PHS-3C, Shanghai, China) a conductivity meter (DDS-307A, Shanghai, China). The ORP is an important driver of microbial community [31], and thus we measured the ORP in laboratory using an ORP electrode (LeiCi 501, Leici Co., Ltd., Shanghai, China), although this measurement probably could not represent the natural soil ORP values since ORP changed rapidly due to oxidation. Soil organic carbon (SOC), total carbon (TC) and soil inorganic carbon (SIC) of homogenized samples were quantified using dry combustion in a TOC analyzer (Vario TOC cube, Elementra, Hanau, Germany), and SOC was determined after carbonate removal [20]. Total nitrogen (TN) was measured using the micro-Kjeldhal procedure with a Foss 8400 Kjeltec analyzer (Foss Analytical Co., Ltd., Höganäs, Sweden). The C:N ratios were calculated from the mass ratio between SOC and TN. Since the samples had different bulk densities, we compared the SOC and TN densities (by multiplying contents with the dry soil bulk densities, with a unit of kg m^−3^).

Total soil DNA was extracted from 0.3 g soil from the samples using the MoBio PowerSoil DNA Isolation kit (MoBio Laboratories, Carlsbad, CA, USA). The extracted DNA was analyzed using a QuBit DNA quantification system (Invitrogen) with QuBit high sensitivity assay reagents. All the soil DNA samples were stored frozen at − 20 °C until further analyses were performed.

### PCR amplification

PCR amplification, purification, and sequencing of a region of the 16S rRNA gene were performed. The V3 and V4 hypervariable region of the bacterial 16S ribosomal RNA gene was PCR amplified (94 °C for 5 min, followed by 35 cycles at 94 °C for 30 s, 54 °C for 30 s, and 72 °C for 30 s and a final extension at 72 °C for 10 min) with the primer set 338F (5′-ACTCCTACGGGAGGCAGCAG-3′) 806R (5′-GGACTACHVGGGTWTCTAAT-3′) by thermocycler PCR system (GeneAmp 9700, ABI, USA). All PCR reactions were performed using a 50 µL reaction volume with 25 µL of PCR Pre-Mixture (TaKaRa), 0.3 µM of forward and reverse primers, and approximately 10 ng of template DNA.

The PCR products and 1X loading buffer (contained SYBR green) were combined and visualized by an agarose gel electrophoresis on a 1.2% (w/v) agarose gel under ultraviolet illumination. PCR products were purified using a QIAquick Gel Extraction Kit (Qiagen, Chatsworth, CA, USA). A single composite sample for sequencing was prepared by combining approximately equimolar amounts of PCR products from each sample. Sequencing was performed (triplicate for each sample) by Majorbio Bio-pharm Technology Co., Ltd. (Shanghai, China) using a Miseq 250.

### Processing of sequencing data

Paired-end reads from the original DNA fragments were merged using FLASH when the original DNA fragments were shorter than twice the length of the reads. The sequences were processed using the QIIME software package Ver. 1.17. The singletons, reads that could not be assembled, were discarded. The chimeric sequences were identified and removed using UCHIME [32], and UPARSE (version 7.1) was used to cluster the operational taxonomic units (OTUs) with a 97% similarity cutoff. To compare of the diversity of bacterial communities in different samples, we estimated the diversity indices including the observed OTUs (which was the count of unique OTUs in the sample), Chao1, Ace, Shannon, and Simpson indices.

### Data analysis

All variable measurements for each soil sample were conducted in triplicate. A one-way ANOVA with a post hoc Tukey’s test was performed to compare the soil parameters and the relative abundance of bacterial phyla from the three permafrost collapse stages using R3.3.1 (http://www.r-project.org/). To test if the sequencing data could be used as indicators for the soil conditions at different stages, we extracted principal components (PCs) from the soil parameters, and then used linear regression analysis to examine the relationships between the PCs and the sequencing data. A detrended correspondence analysis (DCA) showed that the eigenvalue of the bacterial phyla was 1.4, and thus a redundancy analysis (RDA) was selected to explore the relationships between the soil parameters and bacteria phyla. The Mantel test and RDA analysis were performed using Vegan packages [33] in R software. The raw reads were deposited into the NCBI Sequence Read Archive (SRA) database (Accession Number: SRR5420059).

## Results

### Bacterial diversity and community structure

A total of 31,894 OTUs (at the 3% evolutionary distance) were identified based on 10,442 reads for each sample. The Good’s coverage estimator for the OTUs ranged from 0.95 to 0.97 (Table 1), and the rarefaction curves showed that the sequences sufficiently covered the bacterial community diversity in the samples (Additional file 1: Figure S1).

**Table 1:** Estimated OTU richness, diversity indices and sample coverage

	OTU	Ace	Chao	coverage	Shannon	Simpson
C0_10	975	1904	1533	0.96	5.04	0.02
C10_25	913	1907	1490	0.96	4.76	0.04
C25_35	937	1922	1509	0.96	5.20	0.01
C35_50	690	1350	1095	0.97	4.62	0.03
D0_10	1081	2166	1735	0.95	5.33	0.02
D10_25	891	1688	1388	0.96	4.55	0.10
D25_35	910	1574	1341	0.97	5.19	0.02
D35_50	731	1238	1112	0.97	4.89	0.02
S0_10	1136	2051	1778	0.95	5.50	0.01
S10_25	1075	2080	1684	0.96	5.45	0.01
S25_35	1250	2290	1952	0.95	5.79	0.01
S35_50	742	1492	1206	0.97	4.40	0.07

C, D, S means the control, collapsing, and subsided areas. 0–10, 10–20, 25–35, and 35–50 means the soil layers of 0–10 cm, 10–25 cm, 25–35 cm, and 35–50 cm

The OTUs encompassed 45 phyla, 103 classes, 240 orders, 432 families and 717 genera. The bacterial community showed great variations among different samples. The most predominant phyla were Proteobacteria (42.1%), Acidobacteria (26.6%), Bacteroidetes (7.9%), and Chloroflexi (5.3%). Nitrospirae and Actinobacteria accounted for 5.0% and 4.0%, respectively (Additional file 1: Figure S2).

**Fig. 2:**
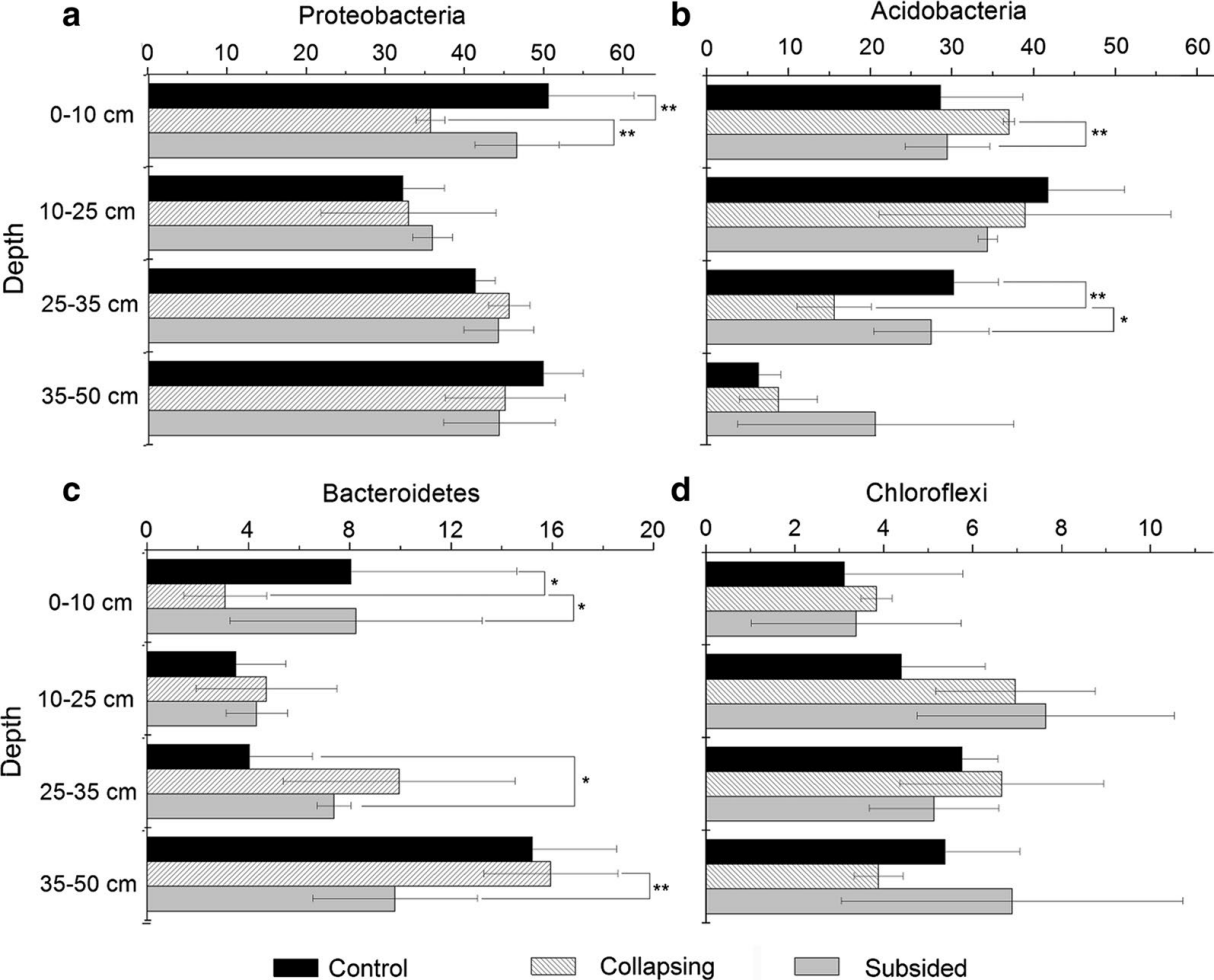
Relative abundance of proteobacteria (**a**), acidobacteria (**b**), bacteroidetes (**c**), and chloroflexi (**d**) for the three stages of permafrost collapse. The significant differences among the three samples were indicated (*p < 0.05, **p < 0.01)

There were markedly changes in the soil bacterial community among the three stages. For example, the Proteobacteria (Fig. 2a) and Bacteroidetes (Fig. 2c) had the lowest relative abundance in the upper 10 cm of the samples in the collapsing areas, while the abundances of Acidobacteria (Fig. 2b) and Chloroflexi (Fig. 2d) were than those in the control and subsided areas.

At the class level, the predominant classes were Acidobacteria (15.2%), Actinobacteria (8.4%), Alphaproteobacteria (10.0%), Betaproteobacteria (8.8%), Deltaproteobacteria (7.7%), and Gammaproteobacteria (4.9%). The latter four classes were recorded in all the samples for the Proteobacteria, but no Epsilonproteobacteria were recorded.

The OTU richness, Ace, Chao and Shannon indices were positively correlated with each other, but negatively correlated with the Simpson index (Table 1). For the mean values of the OTU richness and diversity indices at the four depths, the highest values of OTU Ace, and Chao index were all appeared in the subsided areas (Additional file 1: Table S1).

### Relationships among soil parameters and bacterial phyla

**Fig. 3:**
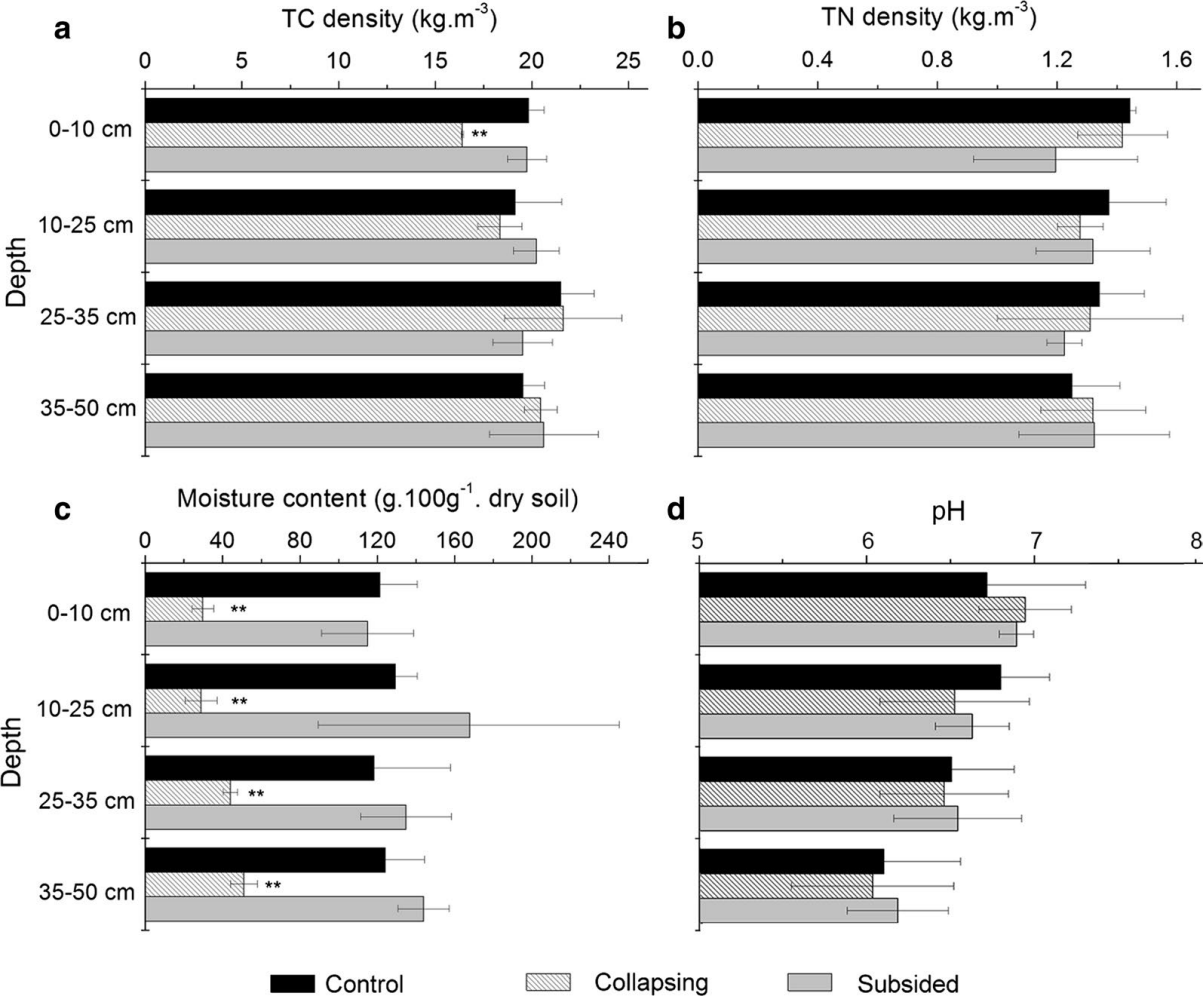
Density of total soil carbon (TC) (**a**), total nitrogen (TN) (**b**), soil moisture content (**c**) and pH (**d**) in different layers for the three stages of permafrost collapse. TC density at 0–10 cm, soil moisture content at all the samples in collapsing areas were significantly lower than those of control and subsided areas (**p < 0.01)

Permafrost collapse obviously changed the soil physio-chemical parameters (Fig. 3). For the upper 10 cm soil samples, TC densities in the collapsing areas were significantly lower than those of control and subsided areas, which probably due to the excessively well-drain condition and severe erosion class. The lowest soil moisture contents were recorded in collapsing soil samples. Soil pH values were generally lower than 7 (Fig. 3).

A Mantel test showed that the bacterial community structure was correlated with pH (r = 0.22, p = 0.007) and depth (r = 0.306, p < 0.001), while other factors, including TC, SOC, SIC, SOC, TN, and C:N ratios, had no significant relationship with the bacterial community structure.

Depth, pH, and ORP was significantly correlated with the relative abundance of specific phyla. TN, TC, and SIC were closely associated with the relative abundance of Nitrospirae. SOC was positively correlated with the occurrence of Bacteroidetes but negatively correlated with the relative abundance of Acidobacteria (Table 2). The most predominant phyla were Proteobacteria, and the relative abundance of Alphaproteobacteria was positively correlated with pH (Pearson’s r = 0.40, p < 0.05) but negatively correlated with the ORP (Pearson’s r = − 0.39, p < 0.05). There was a negative relationship between the relative abundance of Gammaproteobacteria and SOC (Pearson’s r = − 0.35, p < 0.05).

**Table 2:** Relationships among the soil parameters

	Depth	Moisture	Conductivity	ORP	pH	TN	TC	SOC	SIC	C:N
Depth	1									
Moisture	0.061	1								
Conductivity	− 0.068	− 0.226	1							
ORP	0.466**	0.153	− 0.413*	1						
pH	− 0.552**	− 0.064	− 0.060	− 0.533**	1					
TN	− 0.105	0.099	0.259	− 0.402*	0.300	1				
TC	0.281	0.229	0.220	− 0.147	0.011	0.602**	1			
SOC	0.209	0.331	0.203	− 0.128	0.032	0.573**	0.896**	1		
SIC	0.294	0.076	0.190	− 0.135	− 0.013	0.503**	0.893**	0.601**	1	
C:N	0.233	0.127	− 0.129	0.279	− 0.253	− 0.711**	0.047	0.138	− 0.055	1

ORP, oxidation reduction potential (mv); TN, total nitrogen; TC, total carbon content; SOC, soil organic carbon content; SIC, soil inorganic carbon. Pearson’s coefficient, * p < 0.05, ** p < 0.01

### Bacterial phyla as indicators for soil parameters

For all the samples, there were significant relationships between SOC and TN, ORP and pH (Table 3). Five PCs were extracted from the soil parameters. The first and second PCs accounted for 59.97% of the total variances (Additional file 1: Table S2). The first PC (PC1) had highest positive loading coefficients with TC, SOC, and TN, suggesting the organic matter contents were the most important contributing factors for this PC. The most important contributing factors for the second PC (PC2) were moisture content and C:N ratios (Additional file 1: Table S3).

**Table 3:** Relationships between soil parameters and the relative abundance of bacterial phyla

	Acidobacteria	Bacteroidetes	Nitrospirae	Chlorobi	Gemma.	Aminic.	Firmicutes	Caldiserica	Bacteria_un.	Atrib.	Cyano.
Depth	− 0..60**	0.50**	− 0.10	0.68**	− 0.06	0.60**	− 0.13	0.553**	0.45**	0.43*	− 0.25
Mois.	0.06	− 0.00	− 0.15	0.06	− 0.07	− 0.08	0.601**	0.00	− 0.27	− 0.18	0.03
Cond.	− 0.36*	0.41*	0.05	− 0.01	− 0.17	0.25	0.02	0.06	− 0.03	0.33	0.06
ORP	0.09	− 0.21	− 0.17	0.23	0.38*	− 0.01	0.01	0.14	0.36*	− 0.08	− 0.37*
pH	0.45*	− 0.40*	0.22	− 0.50**	− 0.23	− 0.29	0.05	− 0.34	− 0.32	− 0.10	0.14
TN	− 0.04	0.17	0.56**	0.11	− 0.47**	0.19	0.12	− 0.11	− 0.24	0.18	− 0.06
TC	− 0.34	0.33	0.36*	0.30	− 0.21	0.27	0.10	0.02	− 0.06	0.21	− 0.08
SOC	− 0.36*	0.39*	0.22	0.34	− 0.23	0.28	0.30	0.05	− 0.01	0.24	0.03
SIC	− 0.25	0.20	0.43*	0.19	− 0.14	0.21	− 0.12	− 0.01	− 0.09	0.12	− 0.18
C:N	− 0.22	0.09	− 0.45*	0.12	0.35*	− 0.01	0.12	0.16	0.26	− 0.00	0.07

Pearson’s coefficient, * p < 0.05, ** p < 0.01. Moi., Soil moisture content; Cond., Conductivity; Gemma., Gemmatimonadetes; Aminic., Aminicenantes; Bacteria_un., Bacteria_unclassified; Atrib., Atribacteria; Cyano., Cyanobacteria. The proteobacteria was not significantly correlated with these parameters and was not shown

**Fig. 4:**
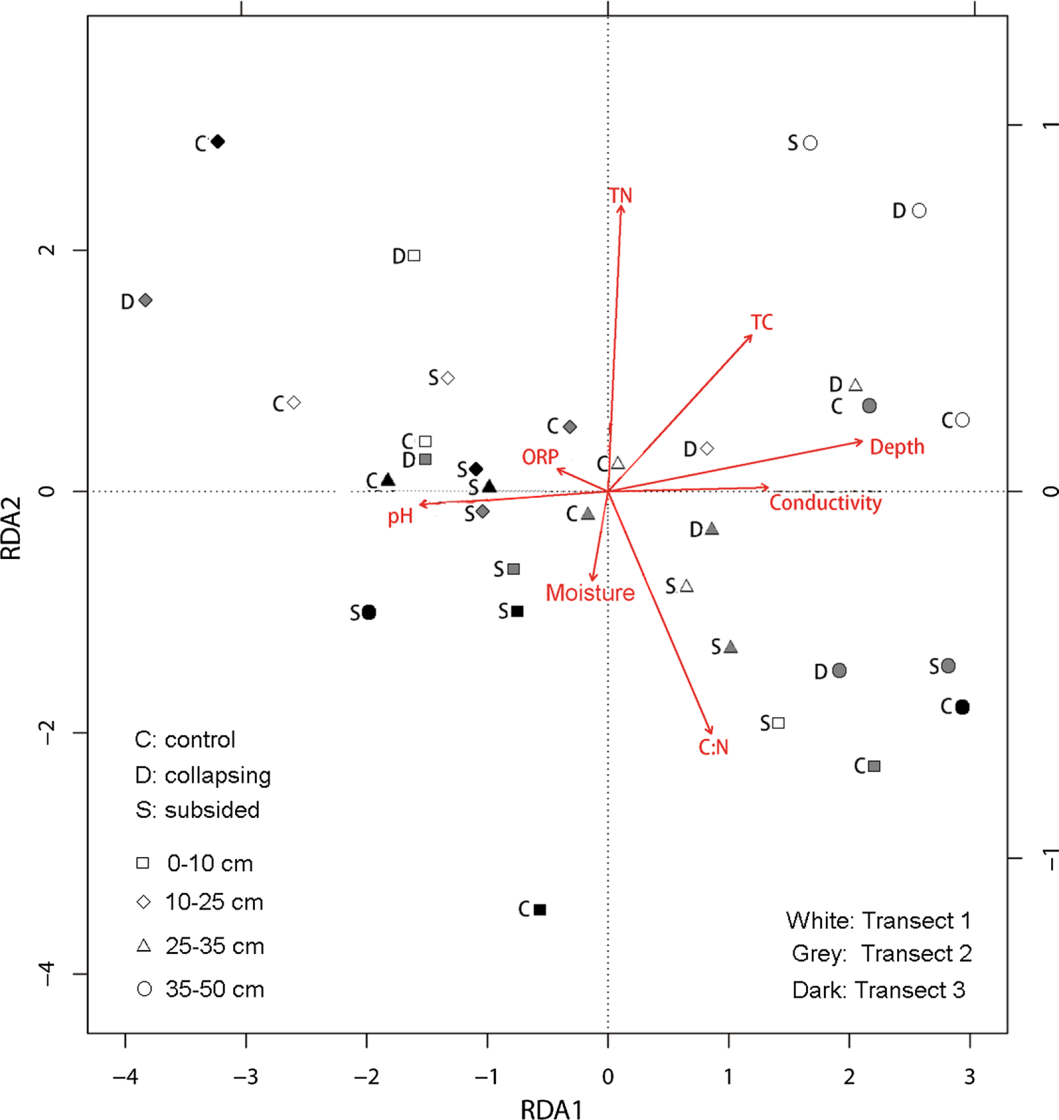
A redundancy analysis (RDA) of the bacterial community structure for the samples from the three stages of permafrost collapse

Linear regressions showed that the PC1 was significantly negatively correlated with the relative abundance of Gemmatimonadetes (Adjusted R^2^ = 0.14, p < 0.02) and Bacteria_unclassified (Adjusted R^2^ = 0.09, p < 0.05). The PC2 was significantly positively correlated with the relative abundance of Firmicutes (Adjusted R^2^ = 0.12, p < 0.05). The RDA results showed that the first and second axes explained 53.0% and 3.0% of the total phyla variances, respectively. The samples of the deeper depths (35–50 cm) were generally scattered in the right part of the RDA chart. The depth and pH were strongly correlated with the first axis. TN content was strongly correlated with the second axis (Fig. 4).

## Discussion

The six dominant phyla accounted for 90.9% of the total sequences, and the number of total phyla (45) were similar with those in the central and southern QTP [19, 34]. The predominant phyla in the present study were similar to most soils irrespective of their climatic conditions [9, 35, 36]. The lower relative abundance of Proteobacteria (Fig. 4a) and the higher abundance of Acidobacteria (Fig. 4b) in the 0–10 cm soil samples in the collapsing area probably can be explained by the lowest organic carbon density at this layer. Proteobacteria tend to grow in soils with high content of labile carbon [37]. In contrast, Acidobacteria usually show higher abundance in soils that have a low-quality carbon supply [37]. Similarly, members of Bacteroidetes (Fig. 4c) are thought to be associated with the degradation of organic matter [38] and thus showed lower abundances in samples in collapsing area. The highest OTUs, Ace, and Chao indices were generally recorded in the subsided areas, which probably showed the effects of labile organic matter contents in the subsided areas. The subsided areas can receive dissolved organic carbon from the surrounding area [14], and the dissolved organic carbon is considered as labile substrates for microbial growth, and thus favors to the increase in soil microbial diversity [39].

The pH and depth were significantly correlated with bacterial community structure in this study. pH is the most important factor driving soil bacterial communities [40–42] since most bacterial taxa exhibit narrow growth tolerances to pH [36]. In subarctic areas, soil depth was one of the most important soil properties influencing bacterial community [8]. In an arid region on the western QTP, the relative abundance of Acidobacteria were higher in the near-surface layers [40]. Although the effects of depth on soil bacterial communities can be partly attributed to the pH because there is a significant relationship between pH and depth, other mechanisms are also probably responsible for the effect of depth, i.e., soil properties varied considerably at the centimeter scale in the upper soil layers [43, 44]. In our study, the relative abundance of Acidobacteria decreased with depth, which can be explained by that most Acidobacteria favor aerobic environments and therefore decline with depth [45, 46].

The Mantel test for all the samples showed no significant relationships among soil carbon and nitrogen and bacterial community. Despite that the smaller sample size at the collapsing stage may have effects on the statistical result, this finding could be also due to the effects of thermokarst on physio–chemical parameters in these samples were uneven. For example, although the SOC density in the upper 10 cm layer in the collapsing stage was lower than that in the control and subsided areas, there were no significant differences at other depths. The non-significant relationship between SOC and bacterial community could be also explained that the SOC consists of many fractions of organic carbon, and the total SOC content was not equal to the substrate availability for bacteria [47].

The effects of ORP on bacterial communities can be partly explained by pH since they were significantly correlated with each other. The significant relationship between Nitrospirae and TN confirmed the fact that higher soil nutrient contents may facilitate the growth of Nitrospirae [45]. It is noteworthy that the relative abundance of Alphaproteobacteria increased along with pH, which was consistent with the finding that this class tended to have a high relative abundance in higher pH conditions [48], but this finding was contrary to one study in arctic region [8]. Since such studies are scarce, future studies are required to understand the relationship between Proteobacterial classes and soil parameters.

The PC1 was significantly negatively correlated with the relative abundance of Gemmatimonadetes and Bacteria_unclassified. The Bacteria_unclassified were the sequences that could not be classified into any known group, and thus the Gemmatimonadetes could be seen as an indicator for this PC. Several studies suggested that Gemmatimonadetes be adapted to a lifestyle associated with organic matter sources that are difficult to mineralize [49]. This is a possible explanation for the fact this phylum was negatively correlated with PC1 in our study. The PC1 was mainly related to higher SOM contents, which usually associated with higher labile fractions [50]. The relative abundance of Firmicutes was a significant indicator for the PC2. Both laboratory experiment [51] and sequencing data from field samples [52] confirmed that this phylum as exhibiting positive response to soil moisture content.

Distribution of sampling sites on the RDA chart showed no clear pattern for the three stages of permafrost collapse, although the deepest layers (35–50 cm layers) tended to appear on the right part of the chart. This result could be explained by the heterogeneity in the soil properties created by thermokarst, and no clear environmental gradient was present at the three stages. Our results showed that permafrost collapse produced a greatly heterogeneous soil environment and subsequently affected the soil bacterial community at the phyla level, and future studies are required to examine the response bacteria at genus and even family levels to environmental changes in thermokarst terrains.

## Conclusions

Our study showed that the loss of organic carbon in the upper 10 cm samples at the collapsing area greatly decreased the relative abundance of Proteobacteria and Bacteroidetes while increased the relative abundance of Acidobacteria and Chloroflexi. The thermokarst increased the bacterial diversity in the subsided areas, a finding that may be related to the presence of higher labile fractions of organic matter. Soil parameters including depth, moisture content, ORP, soil carbon and total nitrogen content showed a significant relationship with specific phyla. The most important factors affecting bacterial communities are depth and pH. The relative abundance of Acidobacteria decreased with depth, but no significant relationships existed between Proteobacteria and depth. Gemmatimonadetes were negatively associated with the factors which were associated with high SOM content, and Firmicutes were significantly associated with soil moisture content and C:N ratios. These two phyla could be used as indicators for the soil parameters in the thermokarst terrain.

## Additional file

**Additional file 1: Table S1.** The mean estimated OTU richness, diversity indices and sample coverage for the four depths. **Table S2.** Principle components extracted from soil variables. **Table S3.** Loading factors for the five principle components. **Figure S1.** The rarefaction curves of the samples. **Figure S2.** Relative abundance of bacterial phyla in soils from the three stages of permafrost collapse.

## Abbreviations

ORP: oxidation reduction potential
QTP: Qinghai-Tibetan Plateau
SIC: soil inorganic carbon
SOM: soil organic matter
SOC: soil organic carbon
TC: total carbon
TN: total nitrogen
