# Supplementary material for: Effects of permafrost collapse on soil bacterial communities in a wet meadow on the northern Qinghai-Tibetan Plateau
Source: BMC Ecol. 2018 Aug 22;18:27. doi: 10.1186/s12898-018-0183-y (PMC6103961; doi:10.1186/s12898-018-0183-y)
Supplement: Supplementary file 1 — Additional file 1: Table S1. The mean estimated OTU richness, diversity indices and sample coverage for the four depths. Table S2. Principle components extracted from soil variables. Table S3. Loading factors for the five principle components. Figure S1. The rarefaction curves of the samples. Figure S2. Relative abundance of bacterial phyla in soils from the three stages of permafrost collapse. [file 12898_2018_183_MOESM1_ESM.docx]

**Additional material for**

**Effects of permafrost collapse on soil bacterial communities in a wet meadow on the northern Qinghai-Tibetan Plateau**

**Xiaodong Wu^1^, Haiyan Xu^2^, Guimin Liu^2^, Lin Zhao^1^, Cuicui Mu^3^**

^1^Cryosphere Research Station on the Qinghai-Tibetan Plateau, State Key Laboratory of Cryospheric Sciences, Northwest Institute of Eco-Environment and Resource, Chinese Academy of Sciences, Lanzhou, Gansu 730000, China

^2^School of Environmental and Municipal Engineering, Lanzhou Jiaotong University, Lanzhou, 730070, China

^3^Key Laboratory of Western China's Environmental Systems (Ministry of Education), College of Earth and Environmental Sciences, Lanzhou University, Lanzhou, 730000, China

*Correspondence to: Xiaodong Wu, E-mail: wuxd@lzb.ac.cn

Email address:

Haiyan Xu, [hyxu12@163.com](mailto:hyxu12@163.com)

Guimin Liu, [liuguimin@mail.lzjtu.cn](mailto:liuguimin@mail.lzjtu.cn)

Lin Zhao, [linzhao@lzb.ac.cn](mailto:linzhao@lzb.ac.cn)

Cuicui Mu, [mucc@lzu.edu.cn](mailto:mucc@lzu.edu.cn)

Table S1 The mean estimated OTU richness, diversity indices and sample coverage for the four depths

|  | OTU | Ace | Chao | coverage | Shannon | Simpson |
| --- | --- | --- | --- | --- | --- | --- |
| Control | 879 b | 1771 ab | 1407 b | 0.96 | 4.91 | 0.03 |
| Collapsing | 903 ab | 1667 b | 1394 b | 0.96 | 4.99 | 0.04 |
| Subsided | 1051 a | 1978 a | 1655 a | 0.96 | 5.29 | 0.03 |

Values in a column with different letters differ significantly (P<0.05, ANOVA

followed by LSD test).

Table S2 Principle components extracted from soil variables

|  | Comp.1 |  | Comp.2 | Comp.3 | Comp.4 | Comp.5 |
| --- | --- | --- | --- | --- | --- | --- |
| Standard Deviation | 1.8312 |  | 1.4296 | 1.0985 | 0.9979 | 0.8604 |
| Proportion of Variance | 0.3726 |  | 0.2271 | 0.1341 | 0.1106 | 0.0823 |
| Cumulative Proportion | 0.3726 |  | 0.5997 | 0.7338 | 0.8444 | 0.9267 |

Table S3 Loading factors for the five principle components

|  | Comp.1 | Comp.2 | Comp.3 | Comp.4 | Comp.5 |
| --- | --- | --- | --- | --- | --- |
| Moisture | 0.335 | 0.542 | 0.67 | 0.309 | -0.181 |
| Conductivity | 0.209 | -0.194 | -0.654 | 0.516 | -0.465 |
| ORP | -0.237 | 0.452 | 0.114 | 0.449 | -0.193 |
| pH | 0.127 | -0.412 | 0.365 | -0.554 | -0.235 |
| TN | 0.46 | -0.227 | 0.215 | 0.311 | -0.232 |
| TC | 0.495 | 0.271 | -0.171 | -0.174 | -0.785 |
| SOC | 0.449 | 0.286 | -0.153 | -0.548 | 0.242 |
| SIC | 0.437 | 0.198 | -0.123 | -0.391 | 0.581 |
| CN | -0.160 | 0.480 | -0.261 | -0.596 | 0.557 |


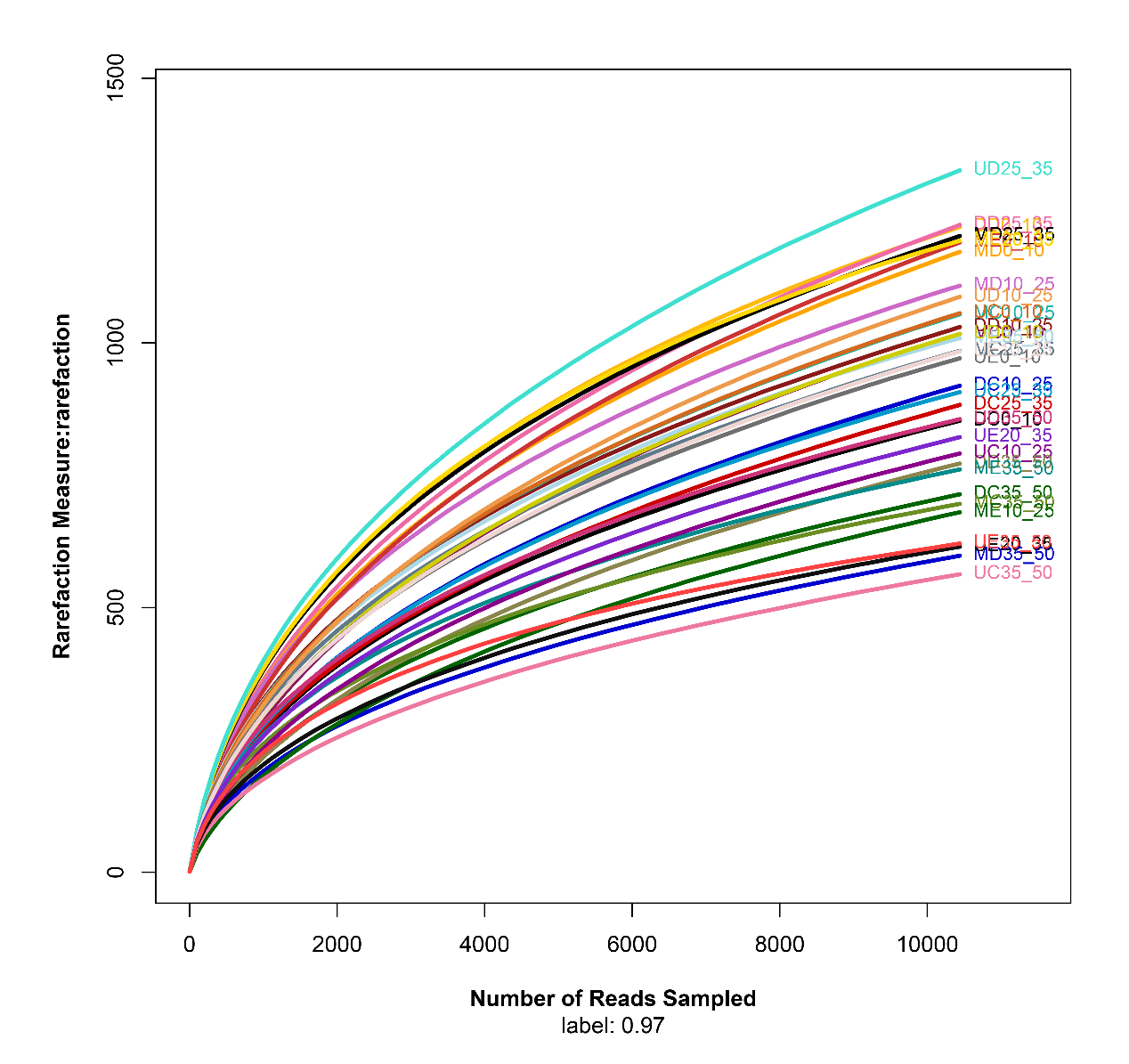


Figure S1 The rarefaction curves of the samples


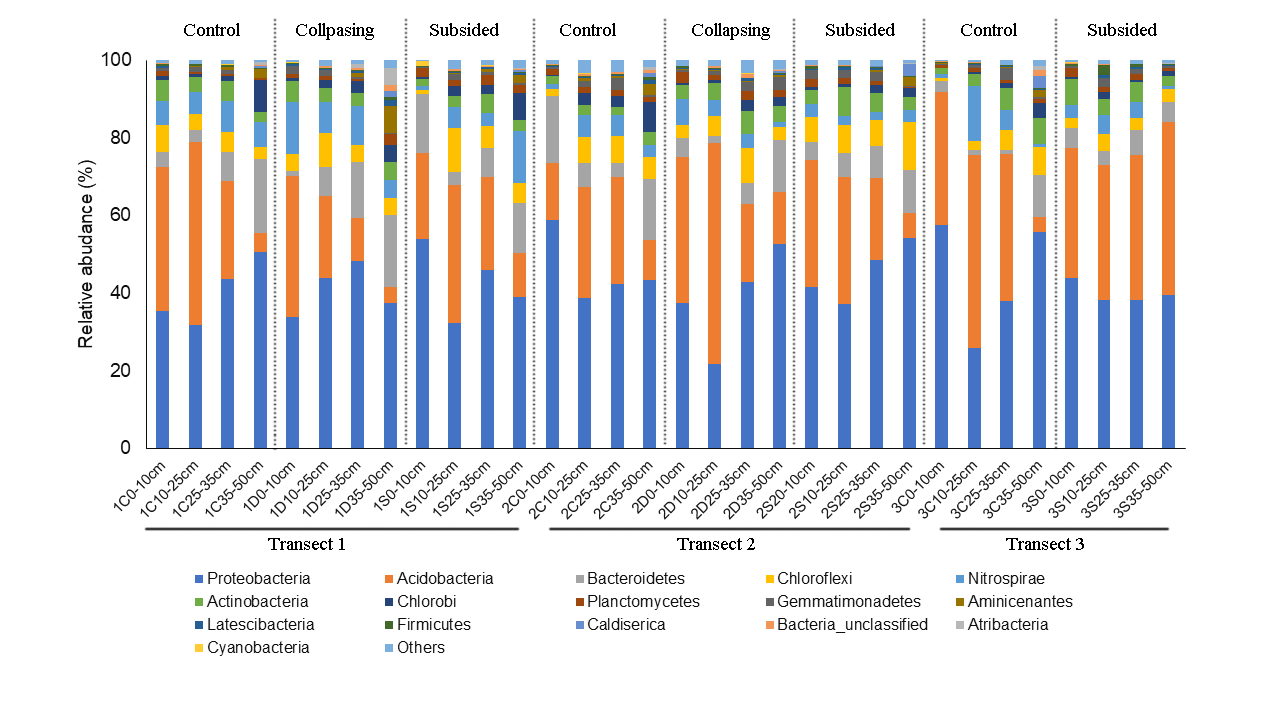


Figure S2 Relative abundance of bacterial phyla in soils from the three stages of permafrost collapse
